# High-purity linearly polarized emission from a compact BIC laser

**DOI:** 10.1038/s41377-026-02322-5

**Published:** 2026-06-23

**Authors:** Yifan Li, Wei Guo, Zihang Cui, Guozhen Liang, Zhongyang Li, Guoxing Zheng, Jin Tao, Zi-Lan Deng, Shaohua Yu, Yongquan Zeng

**Affiliations:** 1 Electronic Information School, Wuhan University, Wuhan, 430072 China; 2 Peng Cheng Laboratory, Shenzhen, 518055 China; 3 Shenzhen Institute of Wuhan University, Shenzhen, 518057 China; 4 State Key Laboratory of Optical Communication Technologies and Networks, China Information Communication Technologies Group Corporation (CICT), Wuhan, 430074 China; 5 Wuhan Institute of Quantum Technology, Wuhan, 430206 China; 6 Guangdong Provincial Key Laboratory of Optical Fiber Sensing and Communications, Institute of Photonics Technology, College of Physics & Optoelectronic Engineering, Jinan University, 510632 Guangzhou, China; 7 Chinese Academy of Engineering, Beijing, 100088 China

**Keywords:** Semiconductor lasers, Nanophotonics and plasmonics, Nanocavities

## Abstract

Bound states in the continuum (BICs) confer ultrahigh quality factors and, thus, enable low thresholds upon photonic crystal (PhC) lasers. However, BICs feature a momentum-space vectorial polarization vortex, posing a substantial obstacle in achieving high-purity polarized emission. Traditionally, polarization control of a PhC laser can be achieved through various symmetry perturbations. However, the emission suffers from low polarization purity due to the finite-size effect inherent in real laser systems. Achieving a high polarization purity in PhC laser emission without significantly sacrificing other performance metrics has thus been a persistent challenge. Here, we develop a dispersion-assisted polarization control strategy for monolithic polarization engineering of PhC laser emission. As a proof of concept, we have demonstrated a linearly-polarized emission from a compact quasi-BIC laser with a high polarization extinction ratio of ~300:1, while single-mode operation and high beam quality are well-maintained. Our work paves a way for efficient polarization tailoring of on-chip lasers. The resulting devices deliver a miniaturized polarization light source for applications across coherent optical communication, advanced display technology, and diverse precision measurements.

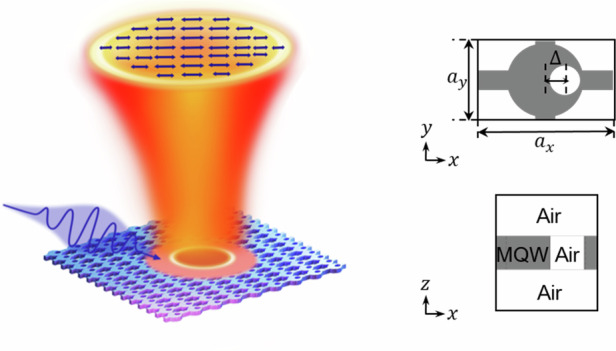

## Abstract (web summary)

We demonstrate a linearly-polarized emission from a compact quasi-BIC laser with a high polarization extinction ratio of ~300:1, while single-mode operation and high beam quality are well-maintained.

## Introduction

Photonic crystal (PhC) lasers, which utilize two-dimensional distributed feedback to realize coherent emission, show promise for high power, good beam quality, and performance scalability^1–3^. Optical bound states in the continuum (BICs) are localized modes with infinite quality (Q)-factor within the radiation continuum, rendering them optimal for single-mode, low-threshold, surface-emitting lasers with even arbitrary spatial polarization profile^4–6^. Merging multiple BICs in momentum (*k*)-space can significantly improve the Q-factor tolerance to structure imperfections and *k*-space deviations^7–9^. Surrounding the BIC structure with band-gap Bragg reflectors^10,11^ or creating a flatband of multiple BICs^12^ provide in-plane light confinement, enabling ultra-low lasing threshold for BIC lasers in micro- or nano-scale size.

Linearly polarized emission, especially in compact forms, holds significant importance across a broad spectrum of cutting-edge applications. These encompass coherent optical communication^13^, single-photon generation^14,15^, advanced bio-imaging techniques^16,17^, and high-dimensional information processing including 3D sensor^18^, volumetric holography^19^, and 3D display technologies^20^. Exploiting the intrinsic optical anisotropy of gain media—such as strained quantum wells^21^, perovskite^22,23^ and organic materials^24,25^—and some polarization-sensitive additives^26–28^ in laser system enables direct generation of linearly polarized light. However, achieving high-purity emission typically necessitates on-chip structural design or external polarizers^25,29^. Polarization control of laser emission in a monolithically integrated feedback cavity is of particular importance as it eliminates the use of external bulky optical components that would also introduce a substantial optical loss and instability. However, the topological singularity nature of BIC and vectorial polarization vortex around the singularity in *k*-space lead to ill-defined emitting polarization state from PhC lasers. One can introduce structural symmetry perturbation to transfer the ideal BIC to a quasi-BIC (q-BIC) with a finite but still high Q-factor in a well-defined linear polarization state at the Brillouin zone center (Γ point)^30–33^. In this way, high-purity linearly polarized emission can be achieved with the assumption of an infinite cavity size^34,35^ (see Section 1 of Supplementary Information). However, the polarization purity is inevitably degraded by the finite-size effect inherent in real BIC laser systems—a critical factor that has long been overlooked in polarization control^36,37^. The finite-size PhC cavity induces momentum quantization, causing the fundamental mode to deviate from the Γ point. Simultaneously, the non-uniform intensity profile of this mode generates momentum uncertainty, which manifests as an out-of-plane divergent beam. Such finite-size effects become particularly pronounced in micro-scale PhC lasers.

In this work, we aim to realize high-purity linearly polarized emission from compact BIC photonic lattices under a paradigm of beam-polarization matching for polarization control. We propose a dispersion-assisted polarization engineering strategy that employs displacement perturbation with broken inversion symmetry for anisotropic polarization control and periodicity deformation with anisotropic lattice dispersion to enhance polarization purity. We systematically investigate the impact of inversion symmetry perturbation strength, lattice dispersion anisotropy, and lattice size on the polarization extinction ratio (PER) of lasing mode in both theory and experiment. The synergistic effects of inversion symmetry perturbation and anisotropic dispersion modulation on the polarization states and emission profiles contribute to significantly enhanced PERs while maintaining a high Q-factor and a good beam quality. Specifically, we realize a single-mode q-BIC laser with high PER (~300:1) in a compact cavity size down to 20 × 20-unit cells (16.4 μm × 13.4 μm).

## Results

**Fig. 1:**
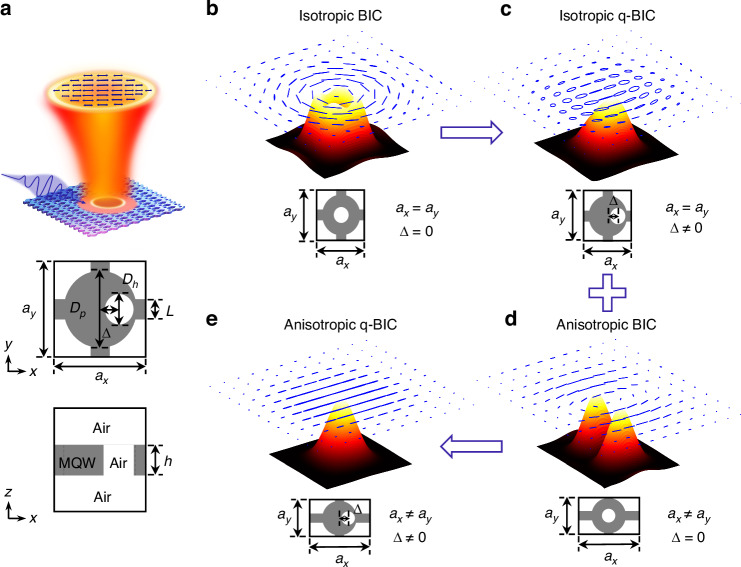
Evolution of bound states in the continuous spectrum (BIC) emission from a compact structure, transitioning from a polarization vortex doughnut to a uniform linearly-polarized beam singlet. **a** Schematic of a photonic crystal (PhC) surface emitting laser emitting a beam singlet with uniform linear polarizations. The horizontal cross-section (*x*-*y*) and vertical cross-section (*x*-*z*) structure profiles of one unit cell of suspended PhC slab are illustrated below. The far-field intensity and polarization profiles for (**b**) a dispersion-isotropic BIC, **c** dispersion-isotropic quasi-BIC (q-BIC), **d** dispersion-anisotropic BIC, and **e** dispersion-anisotropic q-BIC. The major axis of the polarization is proportional to the light intensity. Unless otherwise stated, the structures used in this work have the following geometric parameters: *a*_*x*_ = *a*_*y*_ = 755 nm, *D*_*p*_ = 612 nm, *D*_*h*_ = 242 nm, and *L* = 200 nm for dispersion-isotropic structures; *a*_*x*_ = 820 nm, *a*_*y*_ = 671 nm, *D*_*p*_ = 602 nm, *D*_*h*_ = 240 nm, and *L* = 200 nm for dispersion-anisotropic structures

As shown in the upper panel of Fig. 1a, we consider a finite-size PhC slab composed of multiple quantum wells (MQWs, refractive index ~3.25) surrounded by air. Compared to an infinite periodic lattice, a finite-size cavity induces momentum quantization, causing the fundamental resonance of the surface-emitting mode to deviate from the Γ point. In addition, the electromagnetic field profile in such cavity is subjected to an uneven resonance envelope with gradually attenuated intensity from the center. This degrades near-field periodicity, introduces *k*-space uncertainty, and ultimately generates a divergent beam. In principle, polarization states at specific k-vectors directly correspond to those observed in particular far-field directions. Therefore, high-purity linearly polarized emission requires two critical conditions: (1) spatial homogeneity of the linear polarization state across a broad region of *k*-space (including the Γ-point), and (2) strong overlap between the far-field mode profile and the target polarization state—a principle termed *beam-polarization matching* (Fig. 1a). This requirement is particularly critical for compact BIC lasers, which inherently exhibit large divergence angles. In the following, we reveal that a high-Q BIC state exhibits continuous evolution from a topologically charged polarization vortex to a uniform linear polarization map in *k*-space by deforming a bridged hole-disk structure, an example of which is shown in the lower panel of Fig. 1a. The unit cell has a punched disk with connecting bridges at four sides for mechanical support. To break the inversion symmetry of the square lattice, one can shift the penetrating air hole from disk center along *x*-direction with movement distance (∆) defined as the symmetry perturbation strength. To introduce band dispersion anisotropy, one can set different lattice periods along *x*- and *y*-directions.

**Fig. 2:**
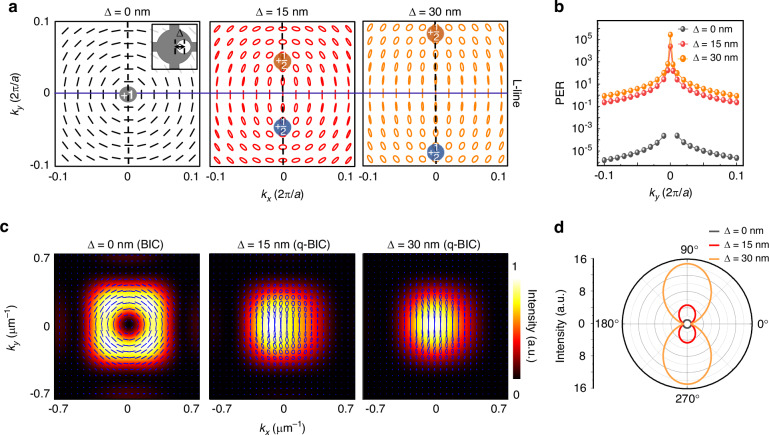
The polarization engineering based on inversion symmetry perturbation for finite-size photonic BIC lattices. **a** Polarization evolution in the momentum space for square lattices with different symmetry perturbation strengths ∆. Insert: a unit cell schematic. The solid circles denote the topological charge and *C*-points. **b** Polarization extinction ratio (PER) evolution along the black dashed lines in (**a**). **c** The far-field intensity and polarization profiles of dispersion-isotropic photonic BIC lattices. **d** Integrated far-field intensity as a function of the polarization angle for dispersion-isotropic photonic BIC lattices. The angle-resolved intensities are normalized to their respective minima, rendering the PER directly accessible from the intensity values. The photonic BIC lattices have a structural size of 20 × 20

A square lattice with *C*_*4v*_ symmetry is well-known to have nearly isotropic band dispersions around a symmetry-protected BIC at Γ point, termed as the isotropic BIC, featuring a polarization vortex and a doughnut-shaped emission beam from finite-size structures (Fig. 1b). To obtain a linearly-polarized emission state, breaking the inversion symmetry (*C*_*2*_) of square lattice is a routine method, which transforms isotropic BIC to an isotropic q-BIC as shown in Fig. 1c. However, the linear polarization emission only occurs along the *L* line (lying in one of the *k*-axis, see Fig. 2a), while the off-*L*-line emitted light exhibits elliptical polarizations. We propose to break the band dispersion isotropy by differentially modulating the *a*_*x*_ and *a*_*y*_ parameters, which appears to be a more effective way to realize linearly polarized emission (Fig. 1d). Notably, this *C*_*2*_-symmetric structure defies conventional intuition by maintaining topological polarization vortex integrity while exhibiting un-defined polarization state as Γ-point. The deliberate dispersion anisotropy induces dual effects: it selectively modifies polarization distributions in *k*-space while reshaping the far-field emission profile, facilitating a better beam-polarization matching. However, this approach inherently produces a two-lobed emission pattern with minimal on-axis intensity along the *y*-direction. To improve the beam quality, we further propose a dispersion-assisted polarization engineering strategy with both symmetry-breaking operation and anisotropic dispersion modulation, as illustrated in Fig. 1e. Such a dispersion-anisotropic structure supports a specific linear polarization state across a large *k*-space region and a q-BIC with a single-lobed far-field beam profile, which is promising for high-purity linearly polarized emission of micro-BIC lasers with good beam quality. Below, we will elucidate the synergistic effects of inversion symmetry perturbation and anisotropic dispersion modulation on the emission profiles and polarization states, and systematically quantify their perturbative strengths on the emission PER of a finite-size BIC structure.

The inversion symmetry breaking perturbs both polarization map in *k*-space and BIC emission beam profile in the far field. The *C*_*4v*_ photonic lattice (*a*_*x*_ = *a*_*y*_, ∆ = 0) exhibits a vortex distribution of linear polarization states around a singular point, featuring an integer topological charge and un-defined polarization state at Γ point (left panel of Fig. 2a). When the inversion symmetry (*C*_*2*_) is broken, a pair of half-charged circular polarization points (*C* points) emerge and shift along the *k*_*y*_-axis from Γ point, following the topological conservation principle of singular optics^38^. In this process, a persistent line of linearly polarized states (hereafter referred to as the *L* line) maintains its orientation independent of the perturbation strength ∆. Concurrently, the major axes of the polarization ellipse at other *k*-points undergo progressive reorientation to align coherently with the fixed linear polarization direction of the *L* line. Figure 2b shows the PERs along the *k*_*y*_-axis (dashed lines) to illustrate the evolution of polarization states. Like the Q-factor distribution of the isotropic BIC band, the PER exhibits a singularity at Γ point and decays fast in the *k*_*y*_ direction. A larger perturbation strength (e.g. ∆ = 30 nm) helps to slightly slow down the PER degradation, but the value is still quite low. Figure 2c superimposes the polarization maps in *k*-space and BIC emission profiles in far field across 20 × 20 finite-size photonic lattices with different perturbation strength. The isotropic BIC emits a vector-vortex beam with doughnut-shaped intensity profile, with its integrated far-field intensity presenting a PER of 1, as indicated in Fig. 2d. In contrast, inversion symmetry perturbation induces a gradual transformation of the q-BIC emission profile toward a Gaussian-like beam. While the *L* line retains linear polarization, off-*L* points exhibit elliptical states that limit the PER. Stronger perturbations yield superior beam quality and linear polarization uniformity; however, they also demand substantial displacement of air holes, which lowers the cavity’s Q-factor. This imbalance establishes a critical trade-off between maintaining the Q-factor and achieving a high PER in the integrated emission.

**Fig. 3:**
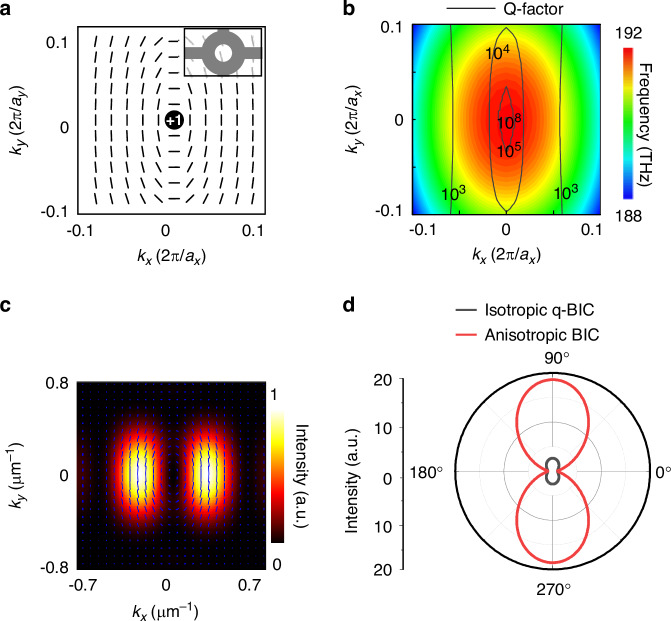
The dispersion engineering based on anisotropic periodicity for finite-size photonic BIC lattices. **a** The *k*-space polarization map of the periodicity-anisotropic photonic BIC lattice. Insert: a unit cell schematic. **b** The dispersion profile of a periodicity-anisotropic photonic BIC lattice with solid contour plots for Q-factor and colored map for frequency. **c** The far-field intensity and polarization profiles of dispersion-anisotropic BIC with a structural size of 20 × 20. **d** The integrated far-field intensity as a function of the polarization angle for dispersion-isotropic q-BIC (∆ = 10 nm) and dispersion-anisotropic BIC with comparable Q-factors

The dispersion anisotropy provides another degree of freedom for emissive polarization engineering, which involves elongating the period along *x*-direction and/or shortening it along the *y*-direction, as shown in the inset of Fig. 3a. The preserved inversion (*C*_*2*_) symmetry generates a polarization singularity at Γ point in *k*-space, which corresponds to a Q-factor extremum. Here, we designate this mode as the anisotropic BIC. The polarization map is dominated by *y*-linear polarization states, while the *x*-linear polarizations are distributed along the *k*_*y*_-axis, which are closely related to the anisotropic dispersion characteristics (solid lines for Q-factor, colors for frequency) shown in Fig. 3b. We can see that both eigen-frequency and Q-factor vary slowly along the *k*_*y*_-axis, while decay relatively fast along the *k*_*x*_-axis. Therefore, the oscillation along the *y*-direction has a lower group velocity and thus a higher Q-factor. On the one hand, the high density of states of flat dispersion along *y*-direction can enhance the light and matter interaction, dominating the optical feedback^39^. On the other hand, for the fundamental oscillating mode in a finite lattice, the mismatch between the optical wavevector and the lattice vector is significant along the *x* direction, contributing to the out-of-plane scattering. Indeed, the anisotropic BIC exhibits a two-lobed far-field profile along the *x*-axis and the polarizations are predominantly oriented along the *y*-axis, while the emission intensity along the *y*-axis with *x*-polarization is significantly suppressed (Fig. 3c). The superior beam-polarization matching leads to a much higher PER than the inversion symmetry perturbation strategy when the two modes have comparable Q-factors in a finite-size structure, as plotted in Fig. 3d. Comparing to inversion symmetry breaking method which always leads to degraded Q-factor, the anisotropic dispersion modulation approach provides a better alternative for linearly polarized light emission with well-preserved high-Q feature.

**Fig. 4:**
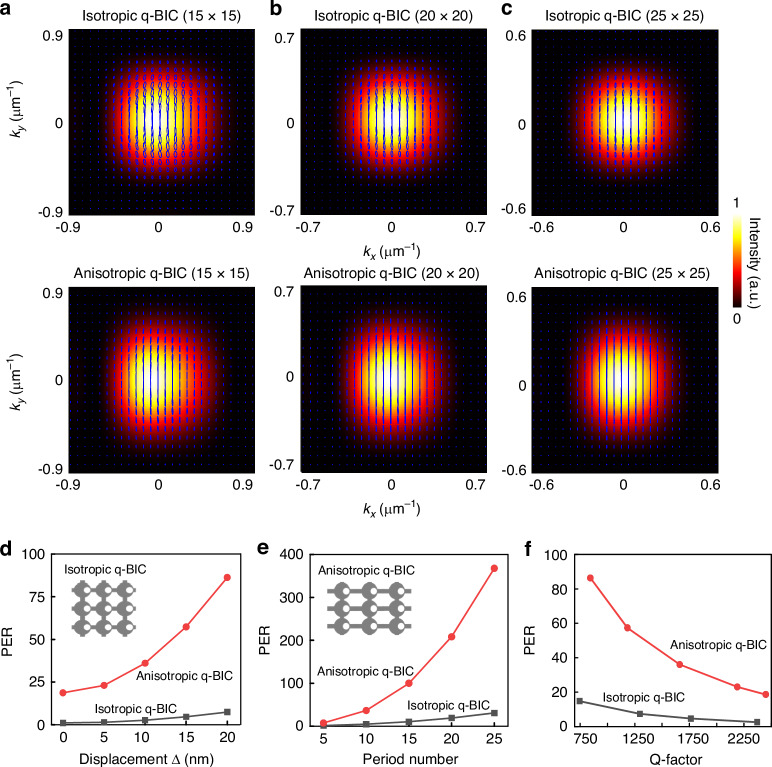
Dispersion-assisted polarization engineering for uniform linearly-polarized emission of finite-size photonic BIC lattices. The far-field intensity and polarization profiles of isotropic q-BIC (upper panel) and anisotropic q-BIC (lower panel) for Δ = 20 nm and structure size (**a**) 15 × 15, **b** 20 × 20 and **c** 25 × 25. **d** The PER of isotropic q-BIC and anisotropic q-BIC as a function of perturbation strength with a fixed photonic lattice size of 20 × 20. **e** The PER of isotropic q-BIC and anisotropic q-BIC as a function of photonic lattice sizes with a fixed perturbation strength (∆ = 35 nm). **f** The PER of isotropic q-BIC and anisotropic q-BIC as a function of Q-factor with a fixed photonic lattice size of 20 × 20. The finite-size isotropic and anisotropic photonic lattices are schematically shown in the insets of (**d**, **e**), respectively

Despite the significantly improved PER of the dispersion-anisotropic BIC, the two-lobed far-field mode profile has a low beam quality. A dispersion-assisted polarization engineering strategy through breaking the inversion symmetry and creating anisotropic band dispersion generates an anisotropic q-BIC state, capable of achieving both high beam quality and homogeneous linear polarizations. The perturbed *C*_*2*_ symmetry eliminates the polarization singularity and leads to prominent light emission along the *y*-axis, while the dispersion anisotropy ensures homogeneous linear polarizations across a large *k*-space region. The polarization-resolved far-field intensity distributions illustrated in Fig. 4a–c provide compelling visual insights into the mechanism underlying the enhanced PER, achieved by scrutinizing the polarization homogeneity throughout the transverse profile of the emitted beam. For example, in Fig. 4a, the two far-field lobes are merged, forming a single-lobed pattern, accompanied by fairly uniform linear polarization states for the anisotropic q-BIC. In stark contrast, the isotropic q-BIC displays pronounced variations in polarization states across the transverse profile. In addition, it can be observed that increasing the overall lattice size can improve the beam quality, producing a nearly circular profile with reduced divergence angle, which further enhances the polarization uniformity. Although the reduced beam divergence also benefits the PER improvement of isotropic q-BIC, the residual elliptical polarization persists at the beam edges even for a 25 × 25 lattice (Fig. 4c).

The interplay among inversion symmetry perturbation strength, dispersion relation, and lattice size significantly influences the PER as illustrated in Fig. 4d–f. Specifically, Fig. 4d shows PER evolution with increasing air-hole displacement (∆) for structures with and without dispersion engineering, both maintained at a fixed array lattice of 20 × 20. The PER of isotropic q-BICs is quite limited although it can be enhanced by increasing the symmetry perturbation strength. In contrast, the aforementioned anisotropic BIC exhibits a substantially enhanced PER value even without deliberate inversion-symmetry breaking. Strikingly, the anisotropic q-BIC exhibits a steep rise in emission PER once minimal air-hole displacement is introduced. These results reveal the synergistic effects of inversion symmetry perturbation and anisotropic dispersion modulation on the PER enhancement of finite-size BIC structures. Figure 4e further presents the consistent performance enhancements through this dispersion-assisted polarization engineering strategy across varying lattice sizes with identical air-hole displacements (∆ = 35 nm). Notably, the anisotropic q-BIC with a compact structure size (15 × 15 lattice) can achieve an exceptional PER of ~100:1, positioning it as an ideal candidate for polarization-controlled micro-laser arrays. In addition, although the dual structural perturbations cause slightly higher cavity losses (see Section 2 in SI), the anisotropic q-BIC has a significantly higher PER than isotropic q-BIC when both states have comparable Q-factors (Fig. 4f). Therefore, anisotropic q-BIC consistently achieves the optimal beam-polarization matching without sacrificing the Q-factors, beam quality, and device compactness.

Achieving high performance in micro-BIC lasers necessitates a delicate balance among the spectral purity, threshold, PER, beam quality, and device footprint, as these performance metrics of the active devices are always interdependent and mutually restrictive. In experiment, the isotropic BIC laser exhibits the lowest lasing threshold with almost isotropic linear polarizations due to the high-Q topological vortex resonant feature (see Section 3 in SI). The anisotropic BIC laser delivers a comparable emission threshold and a prominent polarization anisotropy with PER of 5.7:1 (see Section 3 in SI). When the inversion symmetry perturbation is further introduced, a significant enhancement of PER with good beam quality is anticipated for anisotropic q-BIC at the cost of slightly reduced Q-factor and increased emission threshold. The influence of dual perturbations on lasing threshold and PER performance is discussed in Section 6 in SI.

**Fig. 5:**
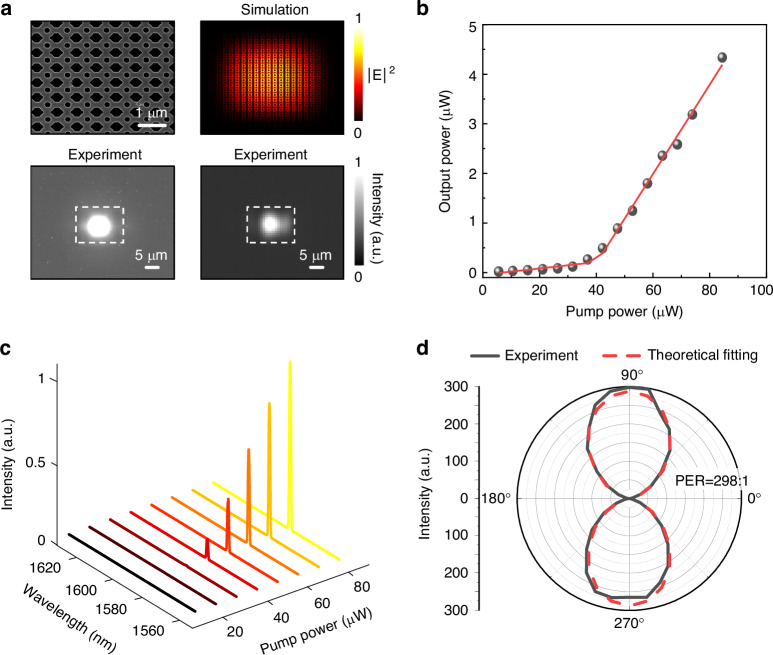
Emission characteristics of the q-BIC laser with dispersion-assisted polarization engineering. **a** The scanning electron microscope (SEM) image of the fabricated device (upper-left panel) with calculated electric field intensity profile (upper-right panel), amplified spontaneous emission pattern (lower-left panel), and photoluminescence above the threshold (lower-right panel). **b** Light-in versus light-out (LL) curve of the anisotropic q-BIC laser. **c** Emission spectra under different pump intensities. **d** Integrated far-field emission intensity as a function of the polarization angle under the pump intensity of ~80 μW, exhibiting a PER of 298:1. The laser has a lattice size of 20 × 20 with following geometric parameters: *a*_*x*_ = 820 nm, *a*_*y*_ = 671 nm, *D*_*p*_ = 602 nm, *D*_*h*_ = 240 nm, *L* = 200 nm, and ∆ = 35 nm

The top view of the anisotropic q-BIC structure with large periodicity anisotropy and significant symmetry breaking (∆ = 35 nm) is shown in the upper-left panel of Fig. 5a. The upper-right panel of Fig. 5a shows a simulated optical field distribution of anisotropic q-BIC mode, exhibiting a more confined intensity envelope and observable electric-field chains in the *y*-direction. This phenomenon can be attributed to the lower group velocity, stronger inter-cell coupling, and thus a higher feedback strength in this direction. The photoluminescence image below the threshold (lower-left quarter of Fig. 5a) shows a near-elliptical emission profile, consistent with the simulation result. And near-field intensity distribution above the threshold is plotted in lower right quarter of Fig. 5a. The nonuniformity in *x*-direction (the slight side lobe on the right) of the laser spots may be induced by the remnant InP buffer layer reflection due to incomplete wet etching. However, the mode is still tightly confined in the *y*-direction. The laser has an emission threshold of ~40 μW (Fig. 5b) and exhibits single-mode operation at a wavelength of ~1590 nm (Fig. 5c). The slightly higher lasing threshold is mainly due to the compact lattice size (20 × 20 lattice, 16.4 μm × 13.4 μm) and the dual symmetry perturbations. In addition, the consistent single-mode lasing is crucial for achieving high PER as the field envelopes of high-order modes are significantly different. By inserting a linear polarizer in front of the spectrometer and rotating polarization angle, the integrated emission intensity exhibits a perfect “8”-shaped polar plot in Fig. 5d. A significant PER value (298:1) is achieved with completely suppressed emission intensity in orthogonal polarization direction. The ultra-high PER and small footprint are promising for advanced imaging, sensing, and display applications requiring high signal contrast and high spatial resolution.

**Fig. 6:**
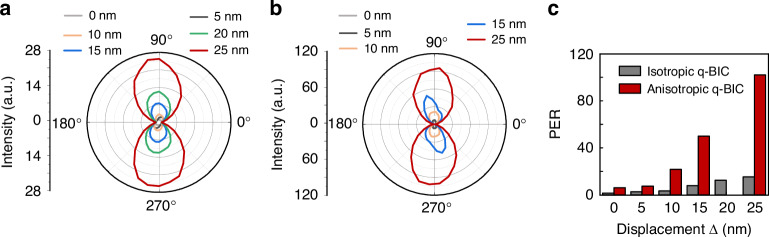
The emissive polarization characteristics of q-BIC lasers with and without anisotropic dispersion engineering as a function of inversion symmetry perturbation strength. **a** Polarization polar plots of isotropic q-BIC lasers with different inversion symmetry perturbation strengths. **b** Polarization polar plots of anisotropic q-BIC lasers with different inversion symmetry perturbation strengths. **c** Comparison of the measured PER values for two types of q-BIC lasers with a finite 35 × 35 lattice

A series of BIC lasers with different symmetry perturbation strengths have also been fabricated. Figure 6a presents the polarization polar plots for isotropic q-BIC lasers. By increasing the inversion symmetry perturbation magnitude by a step of 5 nm from the isotropic BIC case, the PER gradually increases. The polarization polar plots of anisotropic q-BIC lasers are shown in Fig. 6b (the laser structure with ∆ = 20 nm was destroyed during the device fabrication). The anisotropic q-BIC laser has a much higher PER value than that of the isotropic q-BIC counterpart with identical inversion symmetry perturbation strength. Figure 6c compares the PER values of isotropic and anisotropic q-BIC lasers, both showing an increasing trend well-predicted by the simulation results in Fig. 4d. Although anisotropic dispersion modulation generates a notable PER, slight inversion symmetry breaking dramatically promotes this value. This progression compellingly confirms that the highest PER can be achieved through the synergistic combination of inversion symmetry breaking and anisotropic dispersion modulation.

## Discussion

Achieving high-purity polarized emission while preserving the excellent BIC lasing features is challenging for finite-size PhC systems, especially in micro- or nano-scale size. We propose and demonstrate a dispersion-assisted polarization engineering approach based on the principle of beam-polarization matching to realize high-purity linearly polarized emission of BIC lasers. Although either breaking *C₂* symmetry or engineering the dispersion can achieve linearly polarized light emission, applying these approaches in conjunction notably leads to a substantial increase in polarization purity without sacrificing the Q-factor, beam quality, and device compactness. The remarkable synergistic effects lead to an ultra-high PER (~300:1) for a compact q-BIC laser (20 × 20 units, 16.4 μm × 13.4 μm) with consistently single-mode operation and high beam quality. The beam-polarization matching is also essential for engineering the high-purity chiral emission^40–43^. Considering the fact that the polarization control solely relies on symmetry modulations, the dispersion-assisted polarization engineering strategy can be extended to other wavelength ranges and diverse photonic platforms, offering a route to high-performance, monolithically integrated, and miniaturized polarization light sources for a wide range of applications.

## Materials and methods

### Numerical simulations

All band diagrams and eigenmodes were calculated for TE-like polarization with the 3D finite element method simulation software using COMSOL Multiphysics. The method for calculating the polarization map in the momentum space of the infinite structures can be found in ref. ^38^. The far-field intensity distributions of finite structures were obtained through the fast Fourier transform of the near-field intensity distribution. The basic principle of near-to-far-field transformation can be found in ref. ^44^.

### Sample fabrication

The PhC slab was fabricated using an InP epi-wafer consisting of 1 μm thick InP sacrificial layer and 200 nm thick InGaAsP MQWs on the top. The active region, lattice-matched to InP, comprises six In_0.714_Ga_0.286_As_0.863_P_0.137_ quantum wells (emission wavelength 1550 nm) and seven In_0.766_Ga_0.234_As_0.419_P_0.581_ barriers. A hard mask layer with 90 nm SiO_2_ was deposited by using plasma-enhanced chemical vapor deposition (PECVD). Subsequently, Electron-beam lithography (EBL) was performed to create the photonic lattice on a 250 nm polymethyl methacrylate (PMMA) layer coated on the wafer. After development, the pattern was sequentially transferred to the underlying hard mask layer by using CH_4_/H_2_-based reactive-ion etching (RIE) and the MQW layer by ICP-RIE. Finally, the InP sacrificial layer was selectively removed by using a diluted HCl: H_2_O (1:1) solution to form suspended PhC lattices.

### Device characterization

The fabricated devices were characterized by a home-built micro-photoluminescence (µ-PL) measurement system at room temperature (detailed system configurations refer to Section 5 in SI). A 1064 nm fiber laser in pulse mode (9 ns pulse width, 400 kHz repetition rate) was used to pump the laser device in the normal direction with a mask to define the pump profile. The emission pattern on the back-focal plane of the 100× objective lens (NA = 0.7) was imaged onto an InGaAs infrared camera and guided to the entrance slit of spectrometer through three optical lenses. To characterize the PER of laser emission, a rotating polarizer was placed in front of the spectrometer entrance slit. For far-field intensity profile measurement, the camera was placed directly on the Fourier plane by simply removing the Fourier lens. A 50× objective lens (NA = 0.65) was employed to achieve higher momentum space resolution. With a collection angle of ±40°, this objective provides a momentum space range of [−2.63 μm⁻¹, 2.63 μm⁻¹] at 1550 nm. This range fully encompasses all out-of-plane emission wavevectors and enables complete capture of the Fourier plane without artificial truncation. For both near-field and far-field emission pattern measurement, a circular aperture at the entrance pupil of the objective was used to produce an 8 μm-diameter pump spot on the sample. The linear polarizer was also used for polarization-resolved far-field profile characterization.

## Supplementary information

Supplementary Information for High-purity linearly polarized emission from a compact BIC laser

## Data Availability

The data that support the plots of this study are available from the corresponding author upon reasonable request. The codes that support the conclusions of this study are available from the corresponding author upon reasonable request.
