# Supplementary material for: High-purity linearly polarized emission from a compact BIC laser
Source: Light Sci Appl. 2026 Jun 23;15:282. doi: 10.1038/s41377-026-02322-5 (PMC13291335; doi:10.1038/s41377-026-02322-5)
Supplement: Supplementary file 1 — Supplementary Information for High-purity linearly polarized emission from a compact BIC laser [file 41377_2026_2322_MOESM1_ESM.docx]

**Supplementary Information for**

**High-purity linearly polarized emission from a compact BIC laser**

**Yifan Li^1,2,5^, Wei Guo^1,6^, Zihang Cui^1,4,5^, Guozhen Liang^1^, Zhongyang Li^1,4^, Guoxing Zheng^1,2,4^, Jin Tao^6^, Zi-Lan Deng^3,*^, Shaohua Yu^7,*^*,* Yongquan Zeng^1,2,4,5*^**

^1^Electronic Information School, Wuhan University, Wuhan 430072, China
^2^Peng Cheng Laboratory, Shenzhen 518055, China

^3^Guangdong Provincial Key Laboratory of Optical Fiber Sensing and Communications, Institute of Photonics Technology, Jinan University, 510632 Guangzhou, China

^4^Wuhan Institute of Quantum Technology, Wuhan 430206, China

^5^Shenzhen Institute of Wuhan University, Shenzhen 518057, China

^6^State Key Laboratory of Optical Communication Technologies and Networks, China Information Communication Technologies Group Corporation (CICT), Wuhan 430074, China

^7^Chinese Academy of Engineering, Beijing 100088, China

*e-mail: [zilandeng@jnu.edu.cn](mailto:zilandeng@jnu.edu.cn), yush@cae.cn, [yqzeng@whu.edu.cn](mailto:yqzeng@whu.edu.cn).

**Contents**

1. The polarization spectral response of an infinite-size isotropic quasi-bound state in the continuum (q-BIC) structure.
2. The relationship between polarization extinction ratio (PER) and quality (Q)-factor of finite-size BIC lattices.
3. Emission characteristics of isotropic and anisotropic BIC lasers.
4. The polarization-resolved far-field patterns of isotropic and anisotropic q-BIC lasers.
5. The emission linewidth and side-mode suppression ratio (SMSR) of q-BIC lasers
6. The influence of dual perturbations on lasing threshold and PER performance
7. The analysis of beam quality for BIC lasers
8. Reproducibility of dispersion-assisted polarization engineering for q-BIC lasers
9. Experimental setup for BIC laser characterization.

**1.** **The polarization spectral response of an infinite-size isotropic quasi-bound state in the continuum (q-BIC) structure**

**Figure S1** shows the polarization selection rules of an infinite-size photonic crystal (PhC) with A_1_ irreducible representation of *C_4v_* point group at Γ point through inversion symmetry perturbation. **Figure S1a** illustrates an infinite PhC excited by a plane wave from normal direction. The emission polarization of excited resonance is perpendicular to the direction of symmetry perturbation, which have been studied through group theory analysis^1^. **Figure S1b** illustrates the transmission spectra (lower panel) and the corresponding resonant q-BIC profiles (upper panel) for inversion-symmetry perturbed photonic BIC lattice with fixed air hole displacement *∆* = 40 nm and the displacement angle (*θ*) ranging from 0° to 45° and 90° with respect to the *x*-axis. The results clearly show the eccentric air hole destroys the *C_2_* symmetry of electromagnetic field, consequently opening an emission channel. However, the resonant peak disappears when the polarization direction of excitation beam is parallel to the direction of air hole displacement, which implies the mode decoupling to free space in such a polarization state. The independent manipulation of the Q-factor and polarization is revealed in **Figure S1c** and **Figure S1d**. The *y*-polarized transmission spectra of the structures with the air hole moving along the *x*-axis for different displacement distance *∆* are plotted in **Figure S1c.** When the air hole is located at the center of the structure, there is no resonance due to the dark feature of the symmetry-protected BIC. As the *C_2_* symmetry is broken, sharp Fano resonance peaks appear and get broader with the increasing perturbation strength *∆*. However, the structures show no response to *x*-polarized incidence and remain unchanged as asymmetry parameter increases as shown in **Figure S1d.** These results clearly show Q-factor (depends on the perturbation strength) and polarization (depends on the perturbation direction) are fully decoupled in an infinite PhC structure.


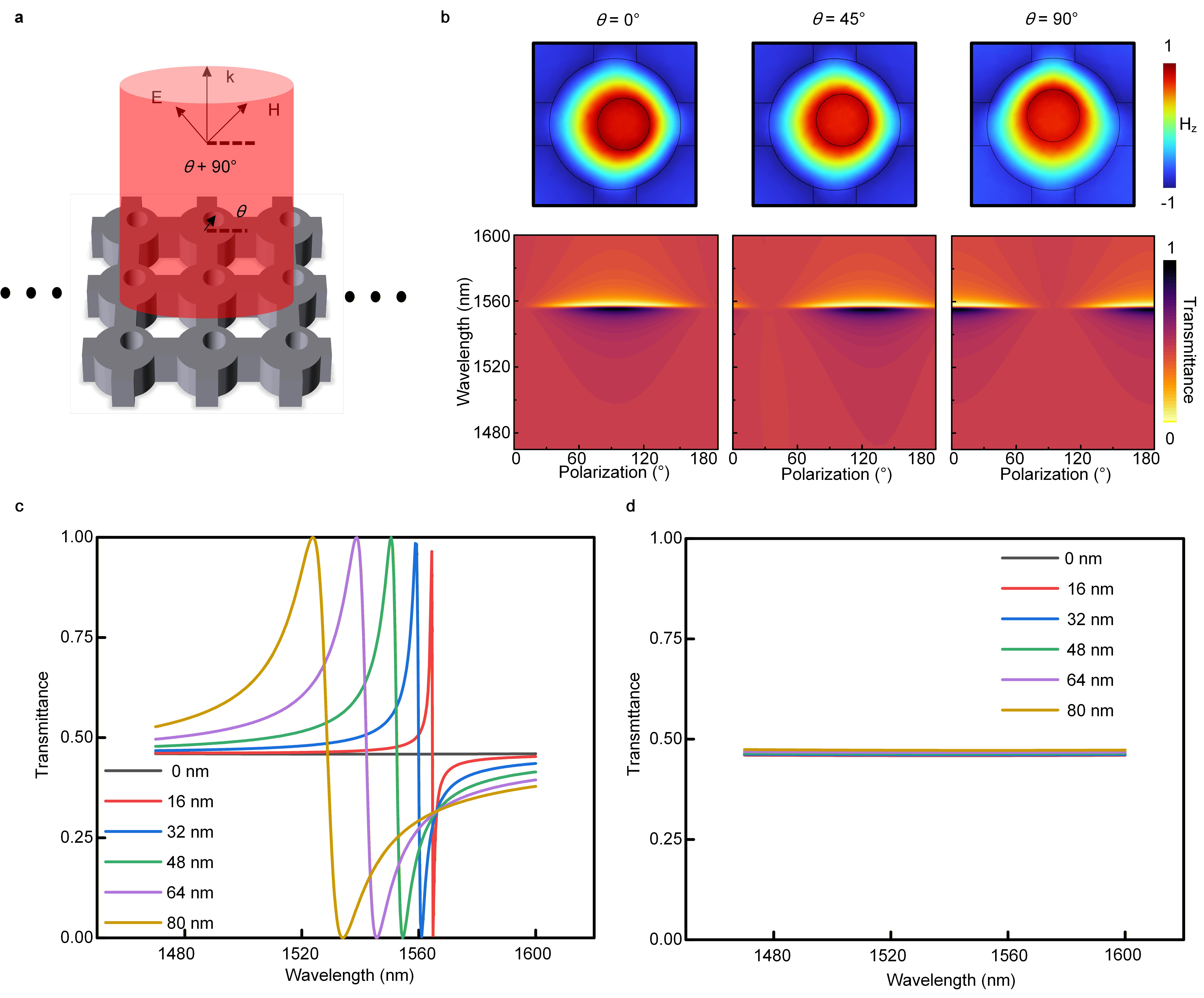


**Figure S1.** **The** **polarization spectral response of an infinite-size quasi-bound state in the continuum (q-BIC) structure. a,** Schematic of plane wave excitation on an infinite-size photonic crystal (PhC). **b,** The transmission spectra (lower panel) and the corresponding resonant q-BIC profiles (upper panel) for inversion-symmetry perturbed photonic BIC lattice with fixed air hole displacement *∆* = 40 nm and the displacement angle (*θ*) ranging from 0° to 45° and 90° with respect to the *x*-axis. **c,** The *y*-polarized and **d,** *x*-polarized transmission spectra of the structures with air hole displaced along the *x*-axis for different distance *∆*.

**2. The relationship between polarization extinction ratio (PER) and quality (Q)-factor of finite-size BIC lattices**

**Figure S2** shows the influence of inversion symmetry perturbation and dispersion modulation on the Q-factors. For a fixed lattice size (the numbers of period are 20 × 20), the Q-factors as a function of perturbation magnitude for the structures with and without dispersion modulation are plotted in **Figure S2a**. It can be observed that for both designs, the Q-factor decreases with air hole displacement. The anisotropic-dispersion modulated photonic lattices exhibit slightly lower resonant Q-factors.  When the symmetry perturbation magnitude is held constant (*∆* = 35 nm), the Q-factor increases with structure size (**Figure S2b**). Structures with dispersion modulation still have lower Q-factors than their unmodulated counterparts. The relationship between the Q-factor and PER is shown in **Figure S2c**. Surprisingly, for a photonic lattice with specific structure size, the anisotropic-dispersion modulation strategy leads to a significantly higher PER than inversion symmetry perturbation approach when the q-BIC states have comparable Q-factors.


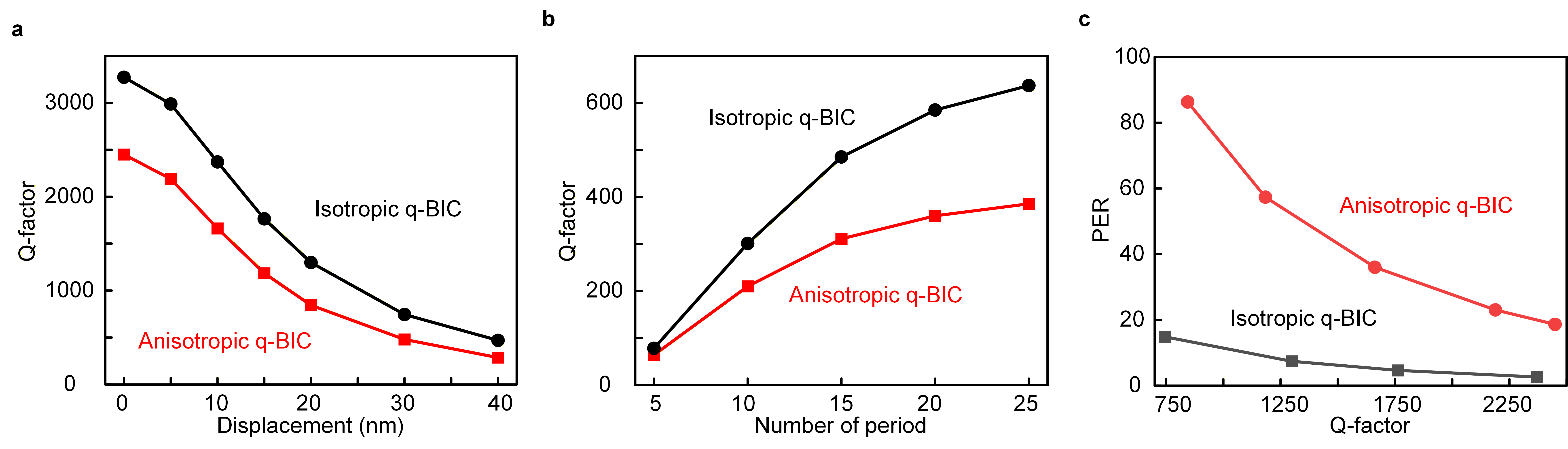


**Figure S2. The relationship between polarization extinction ratio (PER) and quality (Q)-factor of finite-size BIC lattices.** **a,** The Q-factors of isotropic q-BIC and anisotropic q-BIC as a function of the inversion-symmetry perturbation strength. The structure has a lattice size of 20 × 20. **b,** The Q-factors of isotropic q-BIC and anisotropic q-BIC as a function of the photonic lattice size. The inversion-symmetry perturbation strength is fixed to be 35 nm. **c,** The PER as a function of the Q-factor for isotropic q-BIC and anisotropic q-BIC with varied perturbation strengths and a fixed lattice size of 20 × 20.

**3. Emission characteristics of isotropic and anisotropic BIC lasers**

**Figure S3** shows the emission characteristics of the isotropic BIC laser. Different from the structure with dispersion modulation discussed in the main text, the near-field photoluminescence image below the threshold exhibits a circular profile (upper panel of **Figure S3a**), while the emission pattern above the lasing threshold (lower panel of **Figure S3a**) exhibits a doughnut. The light-in versus light-out (LL) curve shown in **Figure S3b** exhibits typical lasing characteristics with a low threshold of ~7 μW. The spectra under different pump intensities are plotted in **Figure S3c,** showing a single narrow emission peak at ~1530 nm. **Figure S3d** presents the integrated far-field intensity as a function of polarization angle under the pump intensity of ~30 μW. The nearly identical emission intensities observed across all polarization angles yield a low PER, a characteristic feature stemming from the topological polarization vortex inherent to the isotropic BIC state. The minor inhomogeneity in polarized intensity can be attributed to spatial variations in the emission profile, potentially arising from the fabrication imperfections.


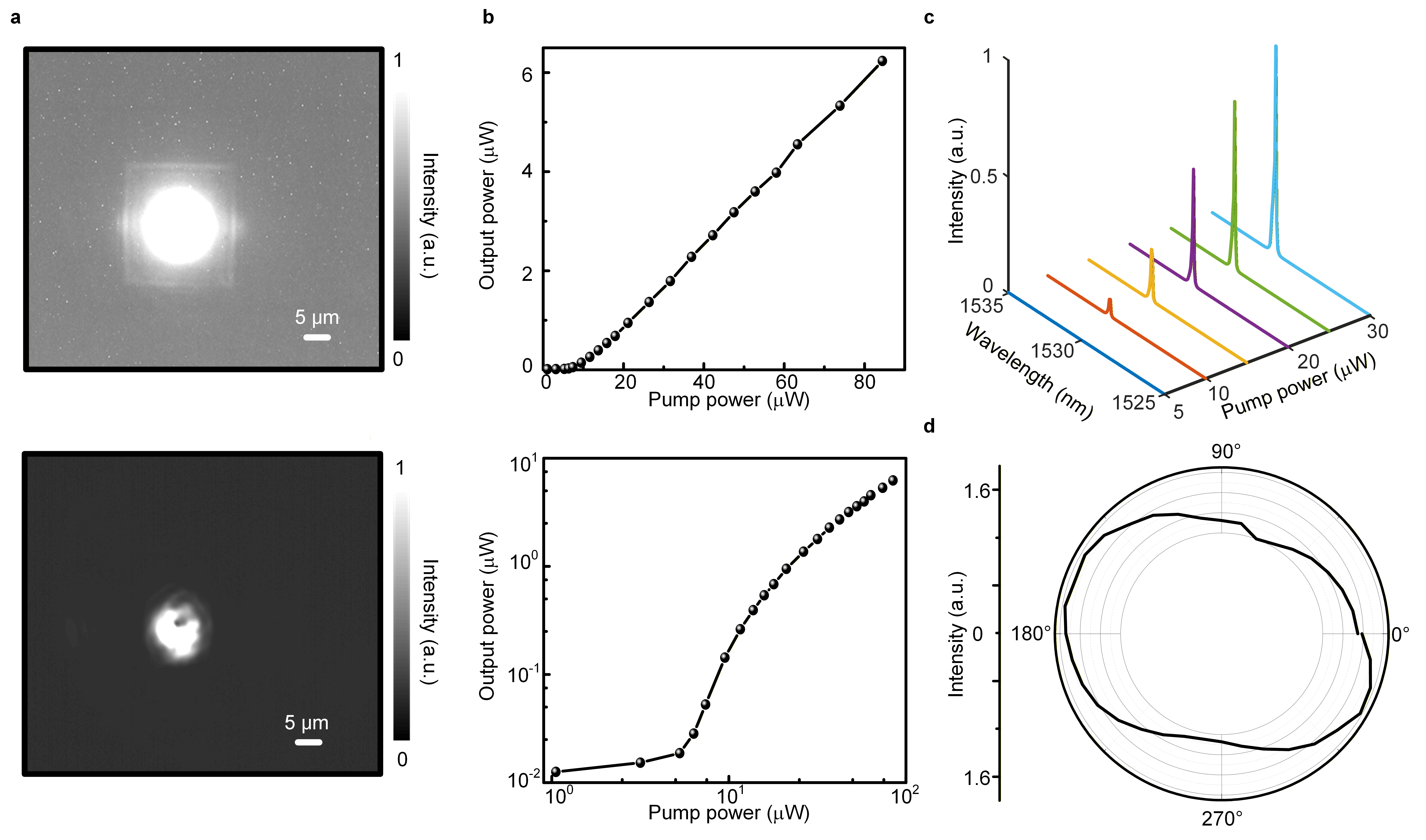


**Figure S3. The emission characteristics of the isotropic BIC laser with a finite lattice size of 35 × 35. a,** The amplified spontaneous emission pattern (upper panel) and the photoluminescence pattern above the threshold (lower panel). **b,** Light-in versus light-out (LL) curve of the isotropic BIC laser in linear *y*-axis scale (upper panel) and semi-logarithmic *y*-axis scale (lower panel). **c,** Emission spectra under different pump intensities. **d,** Integrated far-field intensity as a function of polarization angle under a pump intensity of ~30 μW.

**Figure S4** shows the laser characteristics of the BIC structure with dispersion modulation. The scanning electron microscope (SEM) image of the fabricated laser is shown in the upper-left panel of **Figure S4a**. From the SEM image, it can be observed that the *C_2_* symmetry is well preserved and the period in the *x*-direction is larger than that in the *y*-direction. The upper-right panel of **Figure S4a** shows the calculated intensity distribution of the fundamental mode in the PhC slab, which is more confined in the *y*-direction due to a lower group velocity. The photoluminescence image below the lasing threshold shows an elliptical profile in the lower-left panel of **Figure** **S4a**. The laser emission exhibits a two-lobed pattern in *x*-direction (lower-right panel of **Figure** **S4a**), a typical feature of anisotropic BIC emission. The emission intensity and spectra as a function of pump energy are plotted in **Figure** **S4b** and **Figure S4c**, respectively. These results show lasing action with a threshold of ~9 μW and at the wavelength of ~1570 nm. The threshold is comparable to the isotropic BIC laser (see **Figure S3**), due to the robustness of symmetry protection. And the persistent single-mode lasing shown in **Figure** **S4c** is crucial for maintaining a high PER, as the field envelopes of high-order modes are much more different. Finally, the integrated far-field intensity as a function of polarization angle under different pump strengths are shown in **Figure S4d.** The laser exhibits a stable polarization state above the threshold with obvious polarization anisotropy. The PER is estimated to be 5.7, which is determined by the degree of dispersion anisotropy and the overlapping of far field and polarization map as discussed in the main text.


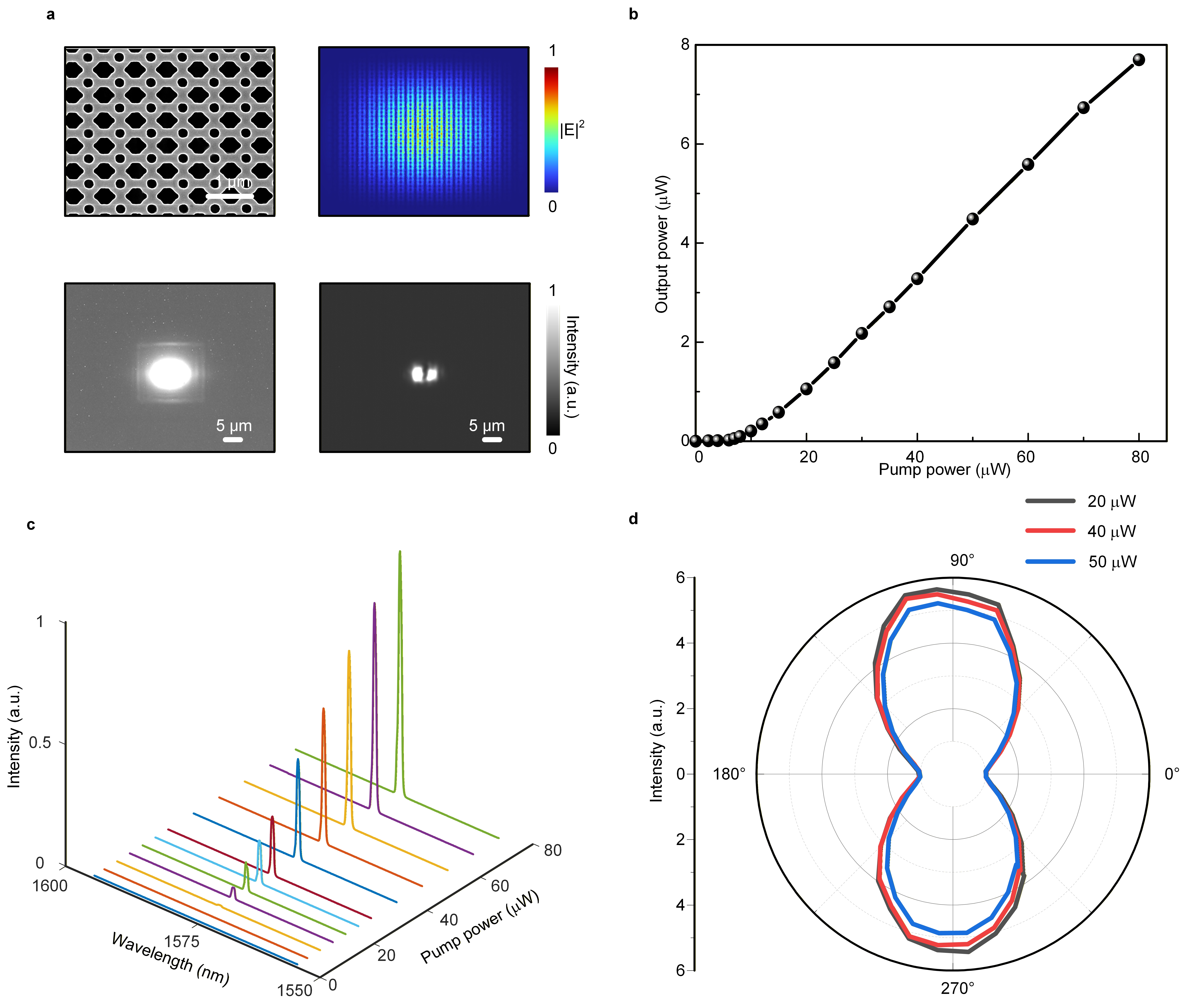


**Figure S4. Emission characteristics of the** **anisotropic BIC laser with a finite lattice size of 35 × 35.** **a**, The scanning electron microscope image of the fabricated device (upper-left panel) with calculated electric field intensity profile (upper-right panel), amplified spontaneous emission pattern (lower-left panel), and photoluminescence emission pattern above the threshold (lower-right panel). **b**, LL curve of the anisotropic BIC laser. **c**, Emission spectra under different pump intensities. **d**, Integrated far-field intensity as a function of polarization angle under different pump strengths.

**4. The polarization-resolved far-field patterns of isotropic and anisotropic q-BIC lasers**

**Figure S5** shows the experimental results of the polarization-resolved far-field images for a micro-size isotropic q-BIC laser with inversion symmetry perturbation, which has a lattice size of 35 × 35 with *∆* = 15 nm. As shown in **Figure S5a**, the laser has a circular emission beam, similar to the simulated result in **Figure 2c**. As theoretically analyzed in the main text, the isotropic q-BIC has linear polarization along *L* line while displaying elliptical polarization at the upper and lower edges. When a polarizer is introduced before the imaging camera and aligned along the *x*-axis (**Figure S5b**), the emission beam shows a two-lobed pattern. In contrast, rotating the polarizer to 45°, 90°, or 135°restores circular beam profiles (**Figures S6c-S6e**). These results validate the far-field beam profile and the polarization map of isotropic q-BIC discussed in **Figure 2c.** Therefore, the isotropic q-BIC laser exhibits a low PER value with inhomogeneous distribution of linear polarization state throughout the transverse beam profile.


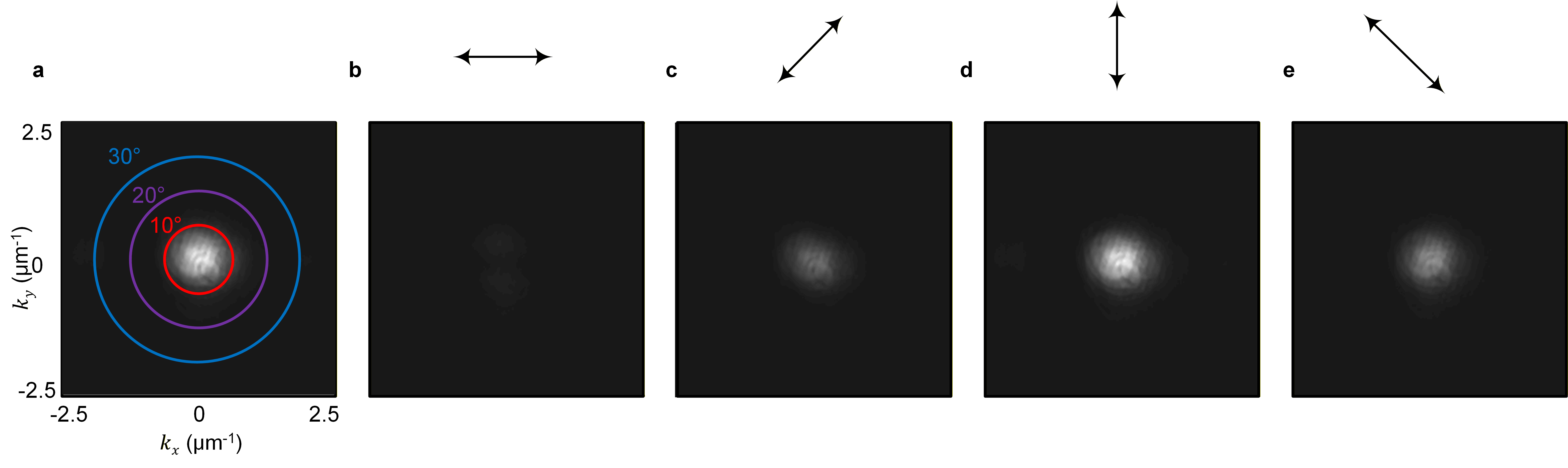


**Figure S5. The polarization-resolved far-field emission patterns of an isotropic q-BIC laser with inversion symmetry perturbation. a**, The far-field emission pattern without an optical polarizer and with the optical polarizer oriented at **(b)** 0°, **(c)** 45°, **(d)** 90°, and **(e)** 135°. The laser has a lattice size of 35 × 35 with *∆* = 15 nm. The arrows on the top indicate the orientation directions of optical polarizer.

**Figure S6** shows the calculated far-filed intensity and polarization profiles of the structure with composite symmetry perturbation and dispersion engineering under different symmetry perturbation strengths. As discussed in the main text and previous sections, the dispersion modulation plays an important role for enhancing PER. However, the optical state has a two-lobed far field if only the birefringence effect is applied as shown in **Figure S6a**, leading to a low beam quality. The beam quality can be improved by introducing symmetry perturbation as shown in **Figures S6b-e**. Once minimal inversion symmetry perturbation is introduced, two far-field lobes exhibit different intensities. As the symmetry perturbation strength increases, the mode evolves from an anisotropic BIC to anisotropic q-BIC with a reduced Q-factor, meanwhile the far-field profile gradually evolves to a single-lobed beam. Meanwhile, the polarization states in between the two far-field lobes gradually become *y*-linear polarized, leading to the higher degrees of beam-polarization matching and larger PER values. Therefore, there is a trade-off between the PER, modal loss, and beam quality for a finite-size photonic lattice.


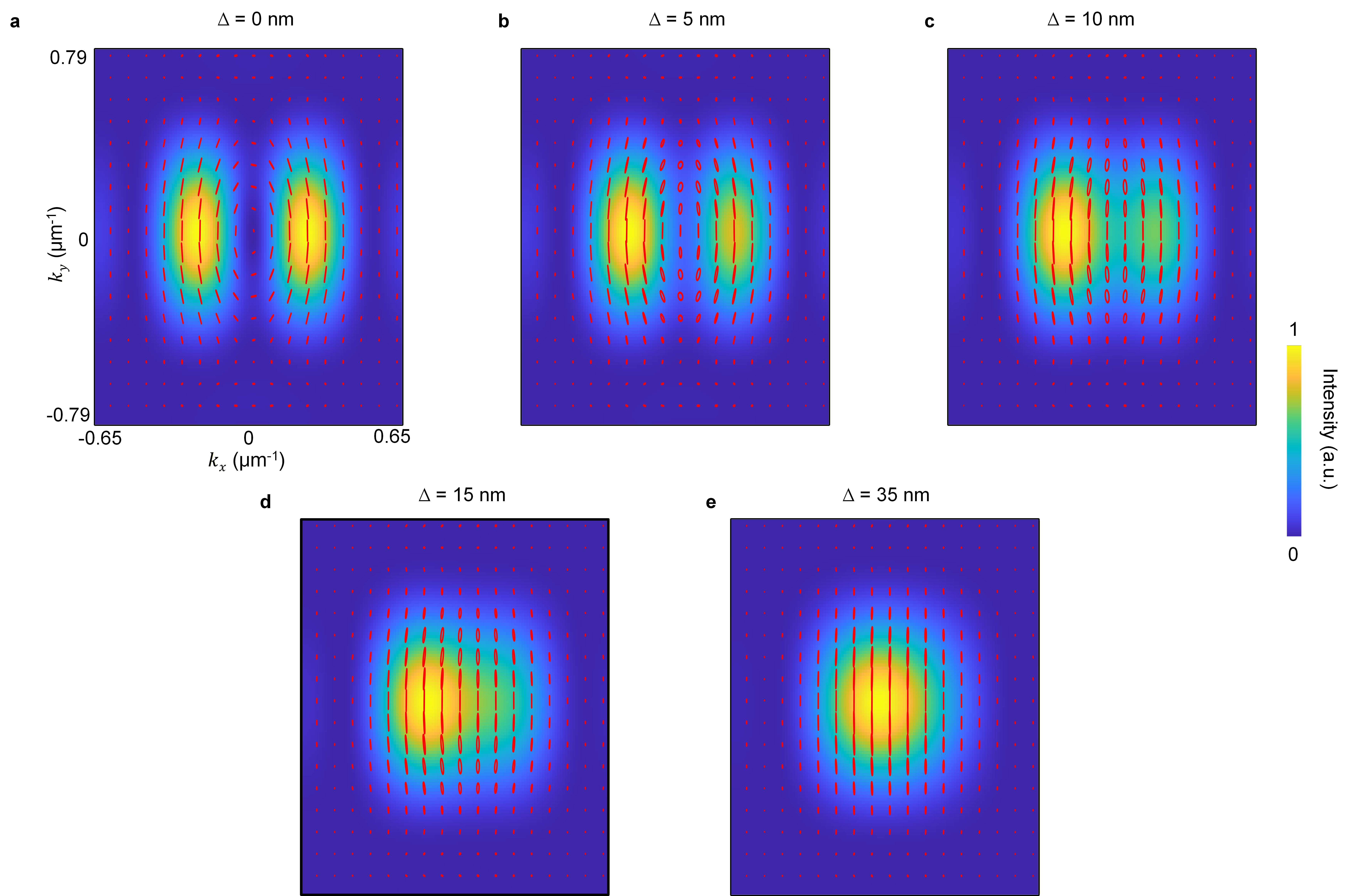


**Figure S6. The simulated far-field intensity and polarization profiles of the structures with dispersion modulation.** **a,** *∆* = 0 nm; **b,** *∆* = 5 nm; **c,** *∆* = 10 nm; **d,** *∆* = 15 nm; **e**, *∆* = 35 nm. The lattices have a fixed size of 20 × 20.

**Figure S7** shows the experiment results of the polarization-resolved far-field patterns for micro-BIC lasers with composite symmetry perturbation and dispersion engineering under different symmetry perturbation strengths. The anisotropic BIC laser (*∆* = 0 nm) exhibits two-lobed far field (**Figure S7a**). When a *y*-axis polarizer is introduced before the imaging camera, the far-field profile is maintained with slightly reduced intensity. The intensity further decreases when the polarizer is rotated to 45° or 135°, and a faint beam can be observed on the noise background when the polarizer is aligned to *x*-direction. These observations confirm both the far-field profile and polarization map of the anisotropic BIC lasing. By breaking the *C_2_* symmetry, the two lobes gradually merge into a single-lobed beam as shown in **Figure S7b-d**. Notably, the *x*-polarized component is completely extinguished when the symmetry perturbation strength is sufficiently large, leading to a high PER.


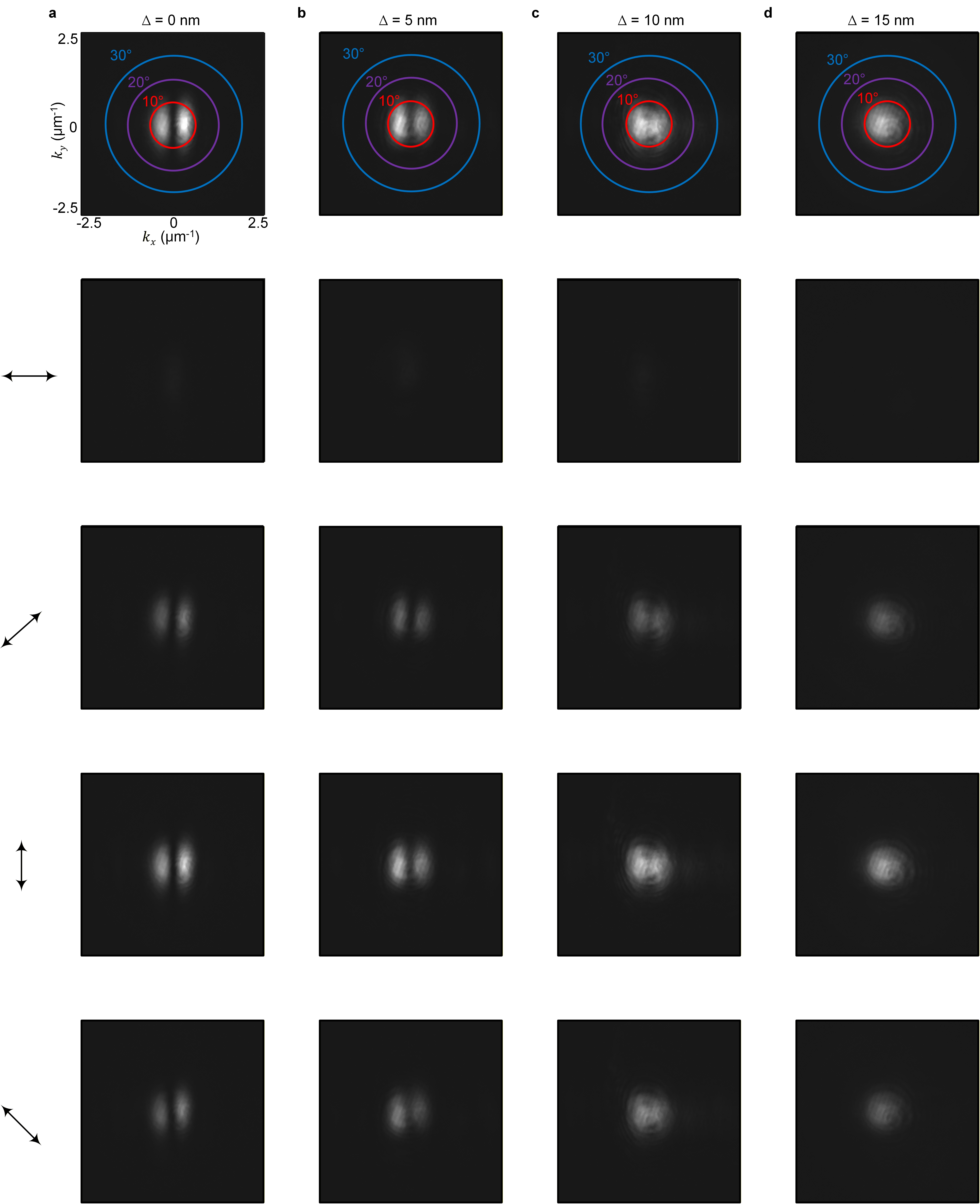


**Figure S7. The polarization-resolved far-field patterns of micro-BIC lasers with composite polarization dispersion engineering.** All the dispersion modulated structures have a lattice size of 35 × 35 with symmetry perturbation of **(a)** *∆* = 0 nm, **(b)** *∆* = 5 nm, **(c)** *∆* = 10 nm, and **(d)** *∆* = 15 nm. The arrows at the left side indicate the orientation direction of optical polarizers.

A high-extinction-ratio linear polarizer is the most direct and sensitive tool for measuring the key metrics—local DoLP and azimuth angle—which are fully determined by the first three Stokes parameters (S_0_–S_2_) as indicated by the Equations (S1-S5).

$S_{0}=I_{0}+I_{90}$ (S1)

$\begin{aligned} S_{1}=I_{0}-I_{90} \#\left( S2 \right) \end{aligned}$

$$\begin{aligned} S_{2}=I_{45}-I_{135} \#\left( S3 \right) \end{aligned}$$

$\begin{aligned} DoLP=\sqrt{S_{1}^{2}+S_{2}^{2}}{/S}_{0} \#\left( S4 \right) \end{aligned}$

$$\begin{aligned} \alpha=0.5\arctan\left( S_{2}/S_{1} \right)\#\left( S5 \right) \end{aligned}$$

By extracting the intensity profiles measured at polarizer angle 0°, 45°, 90°, and 135°, and resorting to Equations (S1-S5), we can derive the spatial profile of DoLP and azimuth angle$\alpha$for isotropic q-BIC/anisotropic q-BIC lasers as shown in **Figure S8**. The results demonstrate that anisotropic q-BIC lasers exhibit more uniform and higher DoLP values, along with a consistent azimuth angle across the beam profile.


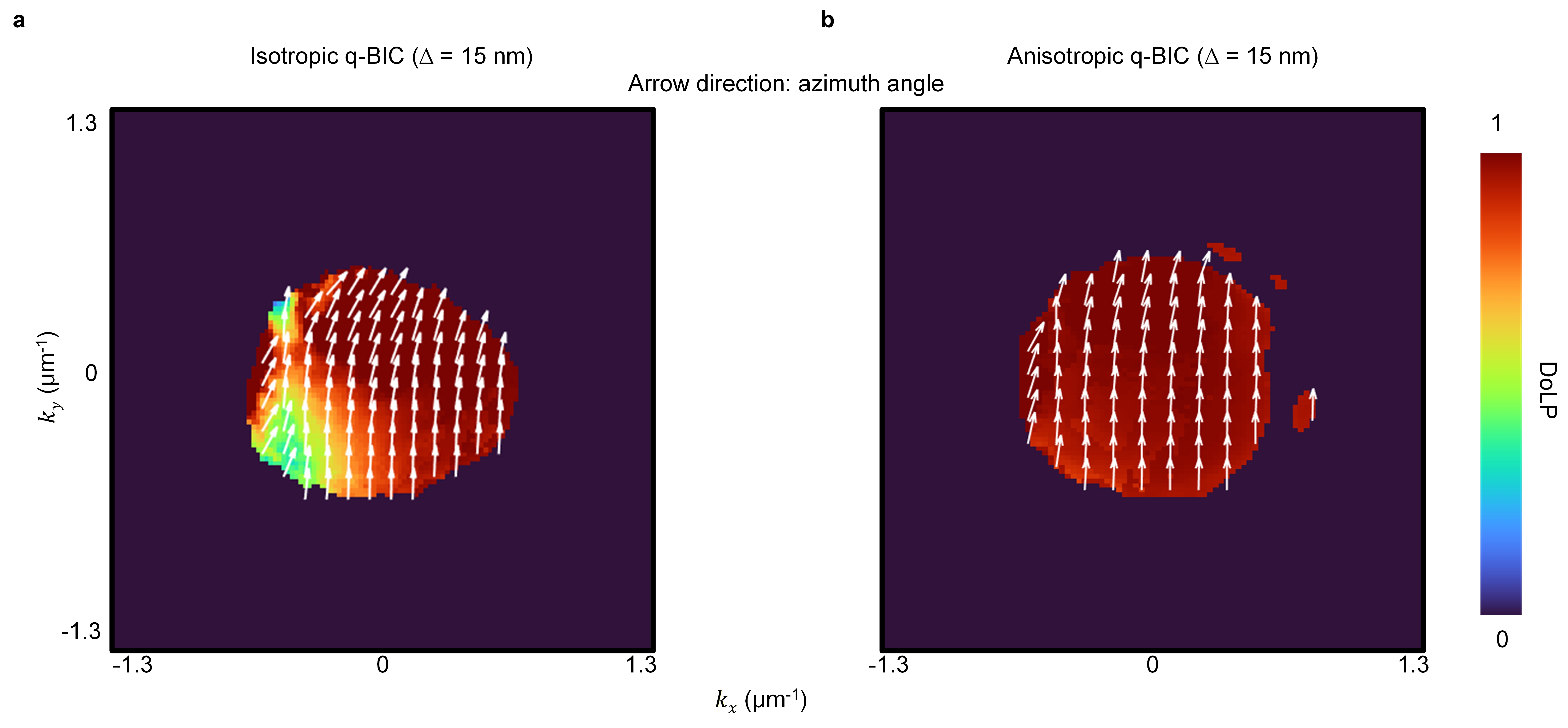


**Figure S8.** **The spatial profile of the local DoLP and azimuth angle of far-field patterns for (a) isotropic q-BIC laser and (b) anisotropic q-BIC laser.** Two lasers have a finite lattice size of 35 × 35.

1. **The emission linewidth and side-mode suppression ratio (SMSR) of q-BIC lasers**

The high polarization extinction ratio (PER) performance is attributed to the robust single-mode operation of the PhC laser, as higher-order modes exhibit much lower PER values compared to the fundamental q-BIC mode. For representative devices of isotropic q-BIC and anisotropic q-BIC lasers with *∆* = 25 nm, we measured high-resolution spectra (spectral resolution: 0.05 nm) and analyzed the linewidth and side-mode suppression ratio (SMSR) across the entire dynamic range. As shown in **Figure S9**, both lasers exhibit abrupt linewidth narrowing near the thresholds, with emission linewidths below 0.35 nm. Spectra acquired immediately above the threshold reveal a single dominant peak and two lasers have a maximum SMSR >38 dB.

In this study, the lasers are optically pumped in pulsed mode. Due to limitations in ultrafast measurement capabilities, all characterization results in this work represent time-averaged data. Nevertheless, the observed high PER and well-defined far-field patterns—consistent with theoretical predictions—indicate stable mode dynamics during a pump pulse.


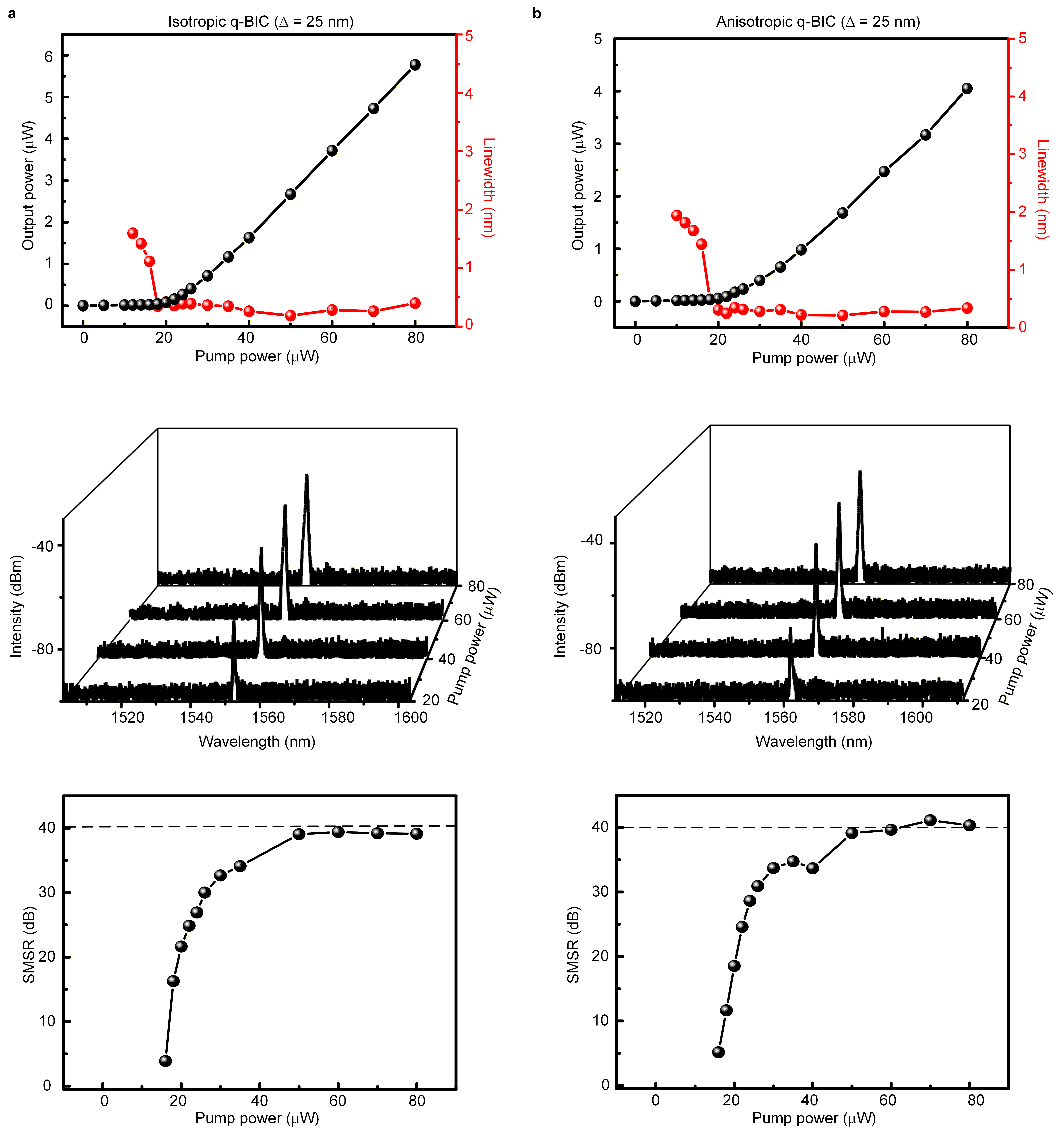


**Figure S9. Emission characteristics of (a) isotropic q-BIC and (b) anisotropic q-BIC lasers with** *∆* **= 25 nm and a structural size of 35** × **35.** Top panels: light-in versus light-out (LL) curves and linewidth vs pump power. Middle panels: emission spectra at various pump powers. Bottom panels: side-mode suppression ratio (SMSR) as a function of pump power.

**6. The influence of dual perturbations on lasing threshold and PER performance**

Displacement perturbation for inversion symmetry breaking induces a higher emission loss and consequently reduce the Q-factor, whereas period modulation for anisotropic dispersion engineering does not affect the emission loss since the operating mode remains a BIC. In **Table S1**, we summarize the lasing thresholds and PER performance for four device types: isotropic BIC laser, anisotropic BIC laser, isotropic q-BIC laser, and anisotropic q-BIC laser. Notably, breaking inversion symmetry apparently increases the threshold, while anisotropic dispersion engineering causes minimal threshold variation. Regarding PER performance, displacement perturbation enhances the PER with a Gaussian beam but at the cost of elevated thresholds, whereas dispersion modulation further enhances PER with negligible threshold degradation. Combining both perturbations enables high PER with good beam quality while maintaining a moderate lasing threshold.

**Table S1**. The threshold and PER performance of various BIC laser designs with a finite 35 × 35 lattice size

| **BIC laser design** | **Threshold** | **PER** |
| --- | --- | --- |
| Isotropic BIC laser | ~7 μw | 1.5 |
| Anisotropic BIC laser | ~9 μw | 5.7 |
| Isotropic q-BIC laser (*∆* = 25 nm) | ~18 μw | 15.293 |
| Anisotropic q-BIC laser (*∆* = 25 nm) | ~18 μw | 102.0776 |

**7. The analysis of beam quality for BIC lasers**

Given the importance of Gaussian beams in laser applications, we evaluate the beam quality through two approaches. (1) Far-field divergence analysis: Since all lasers share identical pump areas and thus similar waist sizes, far-field divergence directly correlates with beam quality $M^{2}$ under the standard definition (i.e., $M^{2}\propto\theta_{\mathrm{divergence}}$for fixed waist size). (2) Gaussian profile fidelity assessment: We fit far-field intensity patterns to ideal Gaussian beam profiles and quantify deviations using root-mean-square error (RMSE):

$$\begin{aligned} \mathrm{RMSE}=\sqrt{\frac{1}{N^{2}}\sum_{x=1}^{N} \sum_{y=1}^{N} \left( I_{kx, ky}-\hat{I}_{kx, ky} \right)^{2}} \#\left( S6 \right) \end{aligned}$$

where $\hat{I}_{kx, ky}$ and $I_{kx, ky}$ denote experimentally measured and Gaussian-fitted intensities at wavevector coordinates $(k_{x},k_{y})$, respectively. This metric provides a rigorous measure of how closely the beam approximates an ideal Gaussian profile.

These methods collectively enable robust evaluation of beam quality despite instrumental constraints. As shown in **Figure S10** (upper panels), the experimentally measured far-field patterns for isotropic BIC, anisotropic BIC, and anisotropic q-BIC lasers reveal that the anisotropic q-BIC laser uniquely achieves a single-lobe Gaussian-like profile with the smallest divergence angle. These patterns are fitted by ideal Gaussian beams (middle panels, **Figure S10**). The beam intensities along *k_x_* and *k_y_* dashed lines indicate that the emission beam of anisotropic q-BIC laser can be better fitted by an ideal Gaussian profile. The lower panels of **Figure S10** shows that the anisotropic q-BIC laser exhibits the smallest RMSE of Gaussian fitting. These results confirm both the superior beam quality and the validity of the beam-polarization matching strategy.


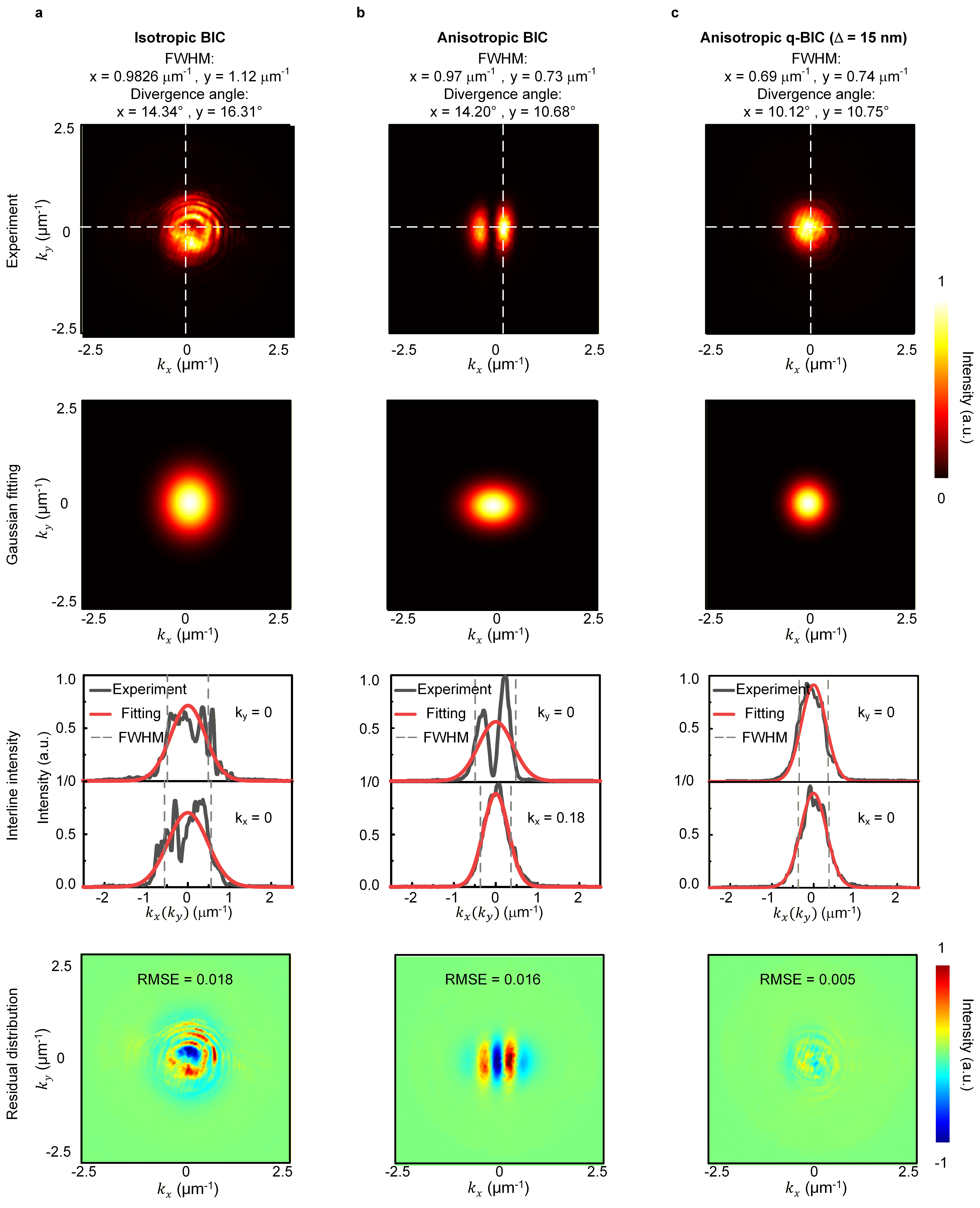


**Figure S10**. **The assessment of beam divergence and the approximation to ideal Gaussian beams**. Measured far-field intensity patterns, ideal Gaussian fits, cross-sectional intensity profiles (along white dashed lines), and residual fitting errors for (**a**) isotropic BIC laser, (**b**) anisotropic BIC laser (*∆* = 15 nm), and (**c**) anisotropic q-BIC laser (*∆* = 15 nm), respectively. All device feature a finite lattice size of 35 × 35.

1. **Reproducibility of dispersion-assisted polarization engineering for q-BIC lasers**

We have conducted comprehensive characterization of an alternative batch of isotropic q-BIC and anisotropic q-BIC lasers (**Figure S11**). Even with two devices exhibiting structural breakage due to fabrication issues, the results corroborate those presented in **Figure 6** of the main text, validating the reproducibility of our findings.


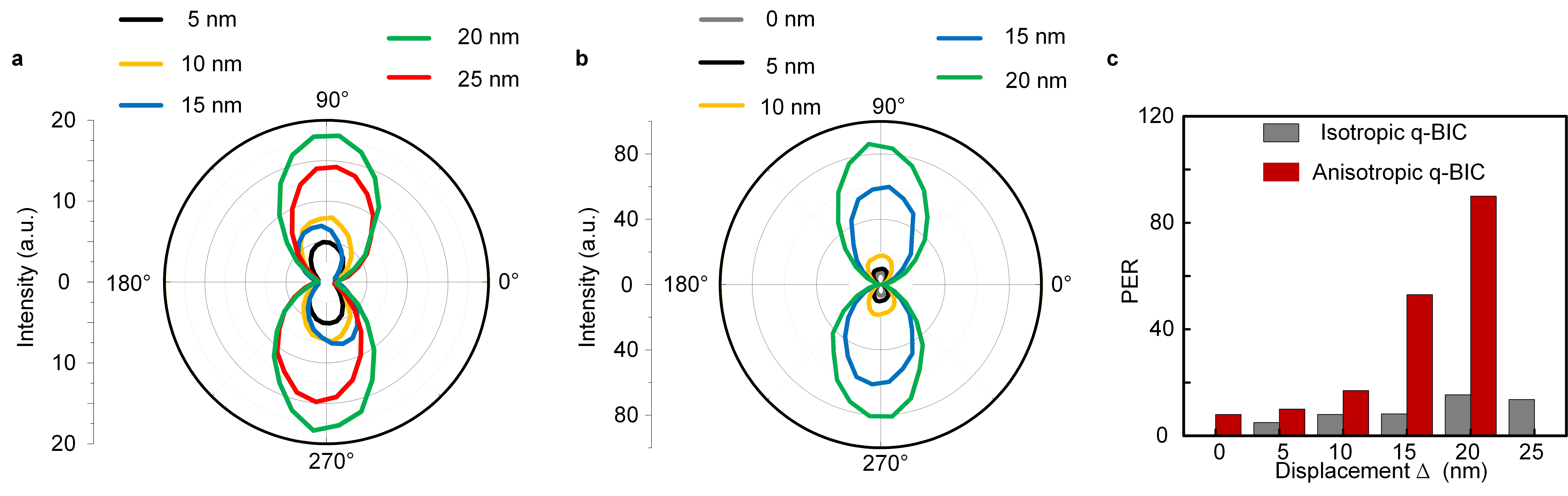


**Figure S11.** **Another batch of q-BIC lasers with and without anisotropic dispersion engineering as a function of inversion symmetry perturbation strength**. **a,** Experimentally measured polarization polar plots for isotropic q-BIC lasers with different inversion symmetry perturbation strengths. **b,** Experimentally measured polarization polar plots for anisotropic q-BIC lasers with different inversion symmetry perturbation strengths. **c,** Comparison of the measured PER values for two types of micro-BIC lasers with a finite 35 × 35 lattice. The geometric structures of isotropic BIC laser and anisotropic q-BIC laser with *∆* = 25 nm were broken during the fabrication.

**9.** **Experimental setup for BIC laser characterization**

**Figure S12** shows the experimental setup for laser emission characterization. A 1064 nm SPI fiber laser in pulse mode (9 ns pulse width, 400 kHz repetition rate, collimated beam diameter 7.5 mm) was used to pump the laser device in the normal direction. The size and the shape of pump light are controlled by a mask. A 50:50 beam splitter and a power meter are used to monitor and stabilize the real-time pump power. A lens with a focal length of 75 mm is used to expand the beam spot and the pump light is focused onto the sample through a 100× objective lens (NA = 0.7). By using the same objective, the emission light on the focal plane of the objective lens is imaged to an InGaAs infrared camera (Xenics Bobcat-320) and guided to the entrance slit of spectrometer (HORIBA Scientific iHR550) through three lenses and a beam splitter. The high-resolution spectra were measured by a spectrum analyzer (YOKOGAWA AQ6370D) with a resolution of 0.05 nm. A rotating analyzer is used in front of the spectrometer to characterize the polarization-resolved emission spectra. For far-field intensity profile measurement, a 50× objective lens (NA = 0.65) was used for higher momentum space resolution and the camera was placed directly on the Fourier plane by simply removing the Lens 2. For both near-field and far-field emission pattern measurement, a circular aperture at the entrance pupil of the objective was used to produce an 8 μm-diameter pump spot on the sample. The rotating analyzer was also placed in front of the camera to characterize the polarization-resolved far-field patterns.


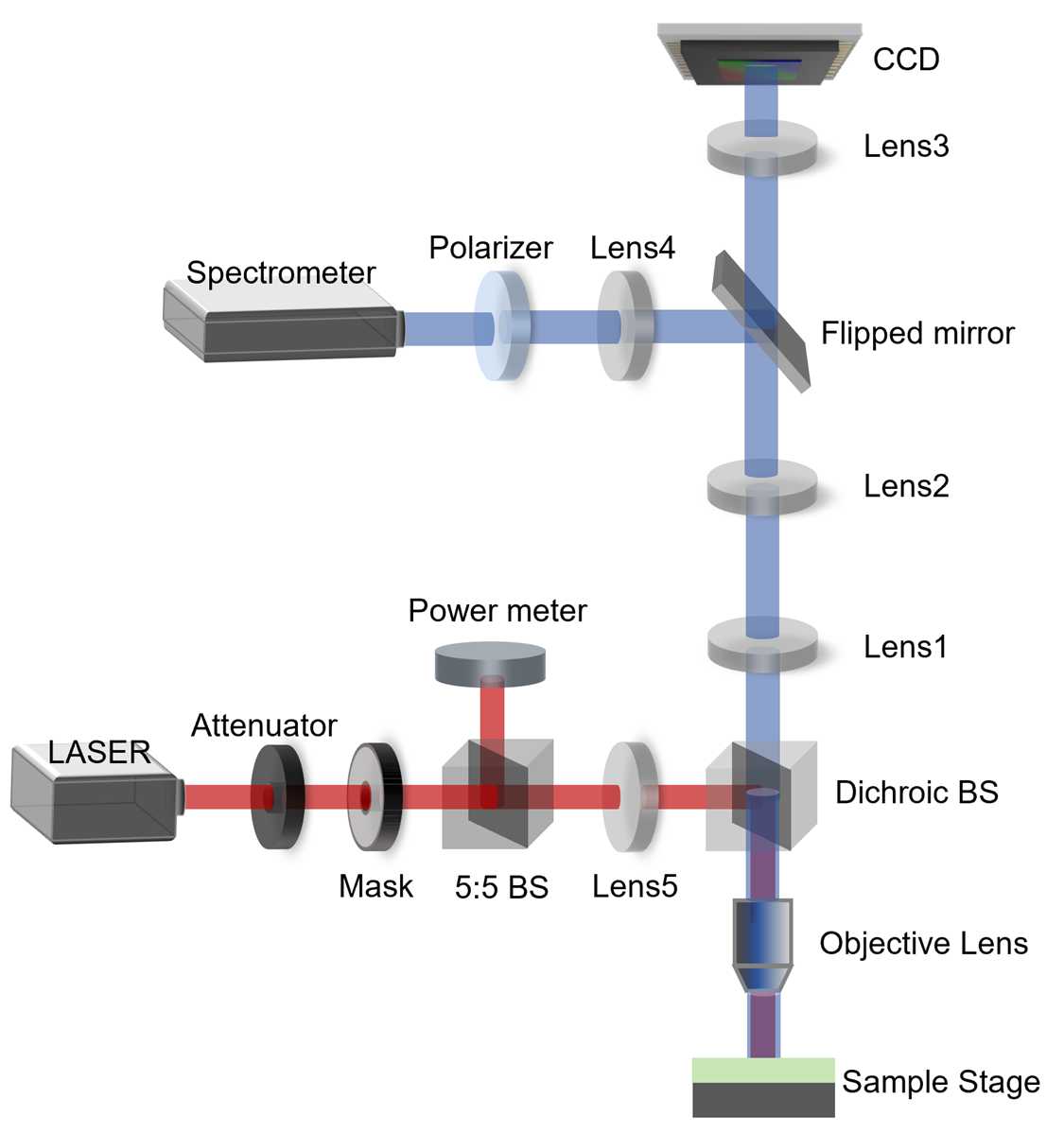


**Figure S12.** **Schematic of the experimental setup for micro-BIC laser characterization.** BS: beam splitter, CCD: InGaAs infrared camera.

**References**

1. Overvig, A. C. et al. Selection rules for quasibound states in the continuum. *Physical Review B* **102**, 035434 (2020).
